# Multiple ophthalmic abnormalities in an infant rhesus macaque (*Macaca mulatta*)

**DOI:** 10.1111/j.1600-0684.2007.00263.x

**Published:** 2008-02

**Authors:** Erin P Ribka, Richard R Dubielzig

**Affiliations:** 1 Division of Veterinary Medicine, Tulane National Primate Research CenterCovington, LA, USA; 2 Department of Pathobiological Sciences, School of Veterinary Medicine, University of WisconsinMadison, WI, USA

**Keywords:** anterior synechia, cataract, hyperkeratosis, optic atrophy

## Abstract

**Background:**

A 4.5-month-old male colony-bred Indian rhesus macaque was presented for presumed trauma to the right eye. The globe was centrally distended 5 mm, with apparent hyphema and moderate to severe corneal edema. Surgical enucleation was performed. The initial histopathology report concluded chronic keratitis. Nearly 3 years later, a diagnosis of goniodysgenesis was made. The eye was sent to a veterinary ocular pathologist for definitive diagnosis.

**Conclusions:**

Final conclusions were anterior segment and corneal defects. Based on the retinal pathology and optic nerve pathology, there was evidence of optic nerve atrophy, a recently described condition in macaques.

## Introduction

Naturally occurring ocular diseases are uncommon in the macaque breeding colonies at the Tulane National Primate Research Center (TNPRC), accounting for only 0.8% of all clinical cases between 2002 and 2006. Thirty-two percent of all ocular abnormalities were secondary to wounding; blepharitis and conjunctivitis accounted for 29% and 21%, respectively, of the remaining ocular abnormalities seen. Corneal ulcers were definitively diagnosed in only 2.9% of all ocular cases. The case described in this report was unusual in the severity of disease. The difficulty in reaching a definitive diagnosis may be, in part, because of the presence of an only recently described abnormality in rhesus macaques, bilateral optic atrophy (BOA). BOA was first described in nine Chinese origin, captive-bred adult rhesus macaques in 2005 [1].

## Case description

**Fig. 1:**
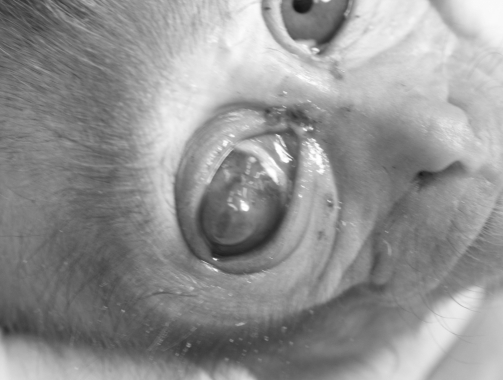
Frontal view, EH86 at presentation.

**Fig. 2:**
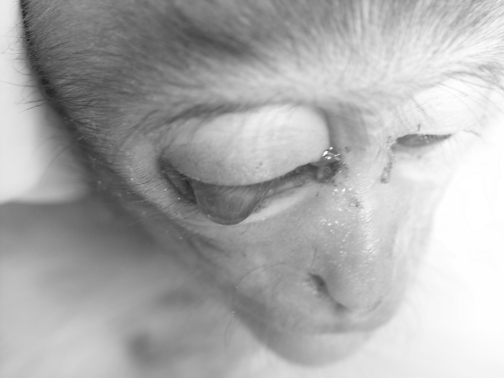
Dorsal view, EH86 at presentation.

On August 15, 2003, a male Indian rhesus macaque (approximate date of birth April 3, 2003) was presented, with his dam, to the breeding colony clinic at the TNPRC for an abnormal right eye (Fig. 1). Initial ophthalmic examination revealed an approximately 3-mm diameter area of the central cornea of the right eye distended an estimated 5 mm, over which the animal was unable to close the palpebrae (Fig. 2). Severe corneal edema was present with possible hyphema in the anterior chamber. An attempt was made to visualize the posterior segment of the globe, but it was not possible with the available equipment and the state of the eye. There was mild chemosis and moderate blepharitis in the right eye. No scleral injection was noted in either eye. The animal was not anesthetized for further examination or diagnostics because of his young age. No abnormalities were noted in the left eye and the remainder of the physical examination was within normal limits. Tonometry was not performed in either eye. Penetrating trauma was suspected. Non-steroidal, triple antibiotic ointment (bacitracin zinc/neomycin sulfate/polymyxin B sulfate) was liberally applied to the affected eye and the animal was given non-steroidal anti-inflammatory drugs (carprofen, 4.5 mg/kg IM twice daily) for pain.

Enucleation surgery was performed on the affected right eye three days after presentation on August 18, 2003. The infant was removed from his dam at 7:00 am and placed in an infant cage with a heating pad and fleece surrogate until surgery. The infant was almost exclusively nursing and additional fasting time was neither necessary nor desirable. He was anesthetized with a mixture of ketamine HCl (10 mg/kg IM) and glycopyrrolate (0.01 mg/kg IM) and an endotracheal tube was placed. Anesthesia was maintained with Isoflurane at 1.5% in oxygen. Buprenorphine (0.009 mg/kg IM) was given at the time of surgery and approximately 8 hours post-surgery for analgesia.

The animal was placed in dorsal recumbency and the area around the right eye was clipped and aseptically prepped with dilute iodine solution. The conjunctiva was incised with scissors and dissected from the sclera periorbitally. The globe was freed from orbital muscles and other intraorbital tissue with metzenbaum scissors. The optic nerve and vessels were severed with scissors and the entire globe removed. Hemorrhage was minimal and was controlled with direct pressure. The lacrimal gland was removed. A periorbital incision was made approximately 3 mm from the palpebral edges and palpebrae excised. Subcutaneous tissues were closed with 3/0 polyglactin 910 (Vicryl, Ethicon) suture in a simple continuous pattern. Skin was then closed with 4/0 nylon (Ethilon, Ethicon) suture in a simple interrupted pattern. The globe was placed in formalin and submitted to the TNPRC Department of Pathology for histopathologic examination. The animal recovered uneventfully and was discharged from the clinic with his dam 8/29/03.

## Histopathology

The initial histopathologic diagnosis was chronic keratitis. Review of one slide 3 years after the enucleation surgery by a veterinarian trained in ophthalmology led to re-evaluation of the case. After a brief evaluation, the veterinarian suspected goniodysgenesis, a condition not described in the literature for rhesus macaques to the best of our knowledge. The paraffin blocks containing the enucleated globe were then sent to the Comparative Ophthalmic Pathology Laboratory of Wisconsin (COPLOW) for further evaluation.

**Fig.3:**
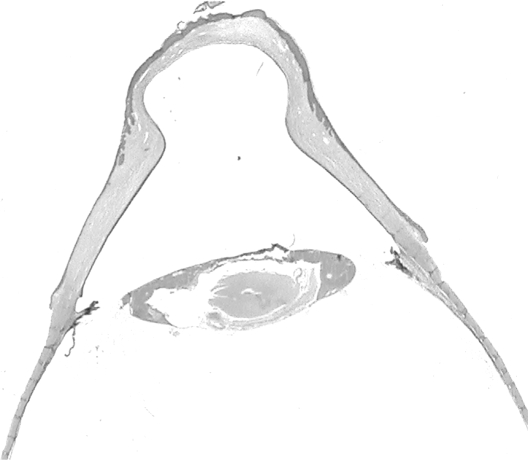
Lens, cornea, and anterior chamber.

**Fig. 4:**
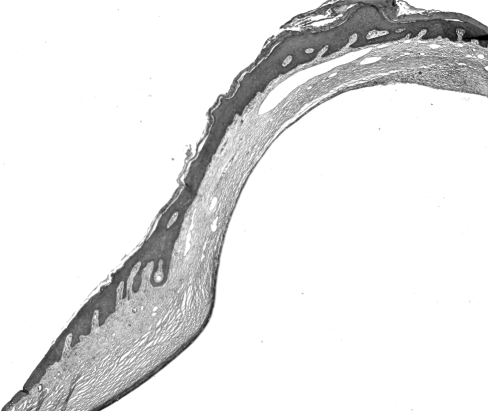
Hyperkeratosis, broad anterior synechia.

**Fig. 5:**
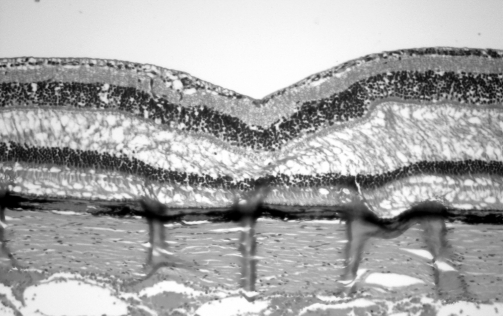
Fovea exhibiting lack of ganglion cells (OA).

**Fig. 6:**
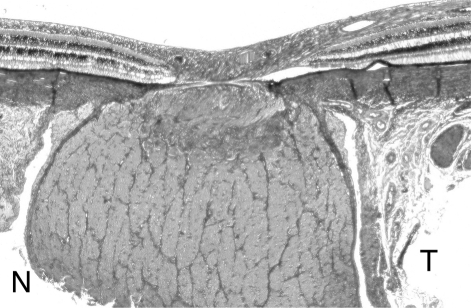
Optic nerve, temporal, and nasal aspects for comparison.

Final histopathologic diagnoses included: corneal hyperkeratosis; broad anterior synechia; axial corneal outpouching with loss of posterior corneal stroma, suggesting a full thickness corneal defect in the neonatal or immediate prenatal period; cataract and possible glaucoma, but there was no evidence of goniodysgenesis in the sections reviewed (Figs 3 and 4). Based on the histologic appearance of the macular retina, and an off center section of the optic nerve, optic nerve atrophy was diagnosed. Optic atrophy was recognized by a greatly reduced number of ganglion cells in the foveal area of the retina, a typical lesion in BOA, and by temporal atrophy of the optic nerve (Figs 5 and 6).

## Discussion

Bilateral optic atrophy is a recently reported condition in rhesus macaques and has been recognized but not reported in cynomolgous macaques also. Histologically, temporal optic nerve axonal loss with minimal gliosis is seen in the optic nerve. There is a variable reduction of ganglion cells confined to the macular retina. There is also thinning of the temporal optic nerve fiber layer adjacent to and extending into the optic nerve head [1]. No behavioral abnormalities were noted in the nine affected animals described. Fundoscopy was used to detect changes associated with atrophy of the optic disk. A flash visual evoked potential demonstrated a decreased amplitude in severely affected animals and a pattern electroretinogram demonstrated changes consistent with ganglion cell loss. There is concern that BOA could have an effect on ophthalmic and visual studies using rhesus macaques. BOA is also a possible non-human primate model of one or several forms of optic atrophy in people. The 2005 paper introducing this interesting disease postulated that the disease has environmental causes or could be inherited in macaques. None of the animals had a history of toxic exposure. Because all nine affected monkeys examined in their study were captive-bred monkeys imported from China, they posited that the disease could be confined to a small population of Chinese origin rhesus macaques. The case presented here could be evidence that BOA is not only confined to this specific group of animals, but also may occur in Indian origin rhesus macaques bred in captivity in the United States.

Regrettably, we were unable to examine the contralateral eye in the animal described in this case report. In September of 2004, he was discovered deceased of unknown causes and the left globe was not recovered. Relatives of this animal still living at the TNPRC will, however, have both globes fixed and examined histologically after death to determine whether his remaining kin share any abnormal ophthalmologic conditions such as BOA or glaucoma.

Bilateral optic atrophy is a recently described condition in rhesus macaques that may have significant effects on research. It may be useful to screen animals for the condition prior to enrollment in visual or ophthalmic studies. With further study and characterization, this condition could be a monkey model of one or several forms of optic atrophy in humans. In addition to describing optic atrophy in an Indian rhesus macaque, this case report serves to remind clinicians to consult specialists when presented with very rare or unusual conditions or diseases. Without the efforts of experts in ophthalmic histopathology at COPLOW, the very interesting diagnosis of optic atrophy would not have been made and a goniodysgenesis screening program might have been prematurely implemented for the Tulane National Primate Research Center rhesus breeding colonies.

## References

[1] Fortune B, Wang L, Bui BV, Burgoyne CF, Cioffi GA (2005). Idiopathic bilateral optic atrophy in the rhesus macaque. Invest Ophthalmol Vis Sci.

